# Informal caregivers of clients with neurological conditions: profiles, patterns and risk factors for distress from a home care prevalence study

**DOI:** 10.1186/s12913-015-1010-1

**Published:** 2015-08-28

**Authors:** Lori A. Mitchell, John Hirdes, Jeff W. Poss, Caroline Slegers-Boyd, Hilary Caldarelli, Lynn Martin

**Affiliations:** Home Care Program, Winnipeg Regional Health Authority, Winnipeg, Manitoba Canada; School of Public Health and Health Systems, University of Waterloo, Waterloo, Ontario Canada; Department of Health Sciences, Lakehead University, Thunder Bay, Ontario Canada

## Abstract

**Background:**

Individuals living in the community with neurological conditions receive the majority of their care from informal caregivers. The purpose of this project was to provide a profile of caregivers of home care clients with neurological conditions. The study also examined prevalence of caregiver distress and the association between neurological conditions and other client and caregiver characteristics with distress.

**Methods:**

The study population included Canadian home care clients in the Winnipeg Regional Health Authority in Manitoba and in the province of Ontario. Clients with RAI-Home Care (RAI-HC) assessment data from 2003 to 2010 were examined. Neurological conditions of interest included Alzheimer’s disease and related dementias, multiple sclerosis, amyotrophic lateral sclerosis, Parkinson’s disease, Huntington disease, epilepsy, muscular dystrophy, cerebral palsy, traumatic brain injury, spinal cord injury, and stroke. Descriptive statistics were analyzed to describe home care client characteristics and caregiver characteristics for each neurological condition. Logistic regression analysis was used to identify risk factors associated with caregiver distress.

**Results:**

A substantial proportion of home care clients were found to have one or more of the neurological conditions (38.8 % to 41.9 %). Caregiver distress was twice as prevalent among caregivers of clients with neurological conditions (28.0 %). The largest associations with caregiver distress were the amount of informal care hours provided in a week and the MAPLe algorithm, an indicator of a client’s level of priority for care. After adjustment for client characteristics, Huntington disease was the neurological condition most strongly associated with caregiver distress. However, clients’ clinical characteristics and informal care hours had a stronger association with caregiver distress than the presence of a neurological condition. Provision of formal home care services provided a protective effect from caregiver distress.

**Conclusions:**

Neurological conditions are common among home care clients and a significant proportion of informal caregivers providing care to these clients experience distress. The complexity of clients with neurological conditions suggests the need for multicomponent support strategies for informal caregivers.

## Background

Many neurological conditions affect an individual’s functioning and result in long-term disability. The World Health Organization (WHO) points to neurological conditions as one of the greatest threats to public health, estimating that the conditions and their direct consequences affect as many as one billion people worldwide. Persons with neurological conditions account for about 6 % of the global burden of disease, and their importance will continue to rise with the aging of populations around the world [1]. The symptoms and disabilities associated with neurological conditions have a major impact on individuals, their families and caregivers, and health service use. Individuals living in the community with neurological conditions receive the majority of their care from informal caregivers, such as family and friends [1–5]. However, when neurological conditions worsen over time, they produce a variety of symptoms and functional impairments that often increase demands on informal caregivers.

Caregivers can experience a high degree of benefit and meaning in providing care, a sense of satisfaction, and a rewarding experience [3, 6–9], though many studies have highlighted the negative consequences of providing care to persons with neurological conditions, including burden and distress. The most cited contributors to caregiver distress are physical demands of care, including support with instrumental and basic activities of daily living (IADLs/ADLs) [10–16]; cognitive impairment [13, 17–20]; behavior disturbances [21–25]; and psychiatric/psychological symptoms (e.g., delusions. depression) [13, 18, 26–28]. Moreover, caregiver distress increases as amount of time spent providing care to recipients with neurological conditions increases [8, 10, 29, 30]. In general, individuals prefer to remain living at home in the community, but to accommodate that preference attention to the informal caregiver is crucial, as caregiver distress may reduce the quality of care and the recipient’s ability to remain in the community [31].

Research to date has not examined and compared the caregiver experience across multiple neurological conditions. One exception, a study by O’Connor and McCabe [31], did look at quality of life for caregivers of people living at home with amyotrophic lateral sclerosis (ALS), Huntington disease, multiple sclerosis (MS), or Parkinson’s disease. The caregivers for people with ALS and Huntington disease experienced greater problems with their quality of life than caregivers for people with MS and Parkinson’s disease. The finding suggested there is merit in comparing and contrasting different neurological groups, rather than examining neurological illness overall, as the needs and experiences of the caregivers for people with different neurological conditions may vary.

The present study is part of the Innovations in Data, Evidence and Applications for Persons with Neurological Conditions (ideas PNC) study, which is preparing an evidence base for the Neurological Health Charities of Canada (NHCC) and the Public Health Agency of Canada (PHAC) on the needs, access to services and interventions, quality of care, and resource requirements of persons with neurological conditions and their informal caregivers (http://www.phac-aspc.gc.ca/cd-mc/nc-mn/index-eng.php). This study focuses on clients receiving public home care to augment the neurological research on the caregiving experience while providing insight into the care needs of a complex population with expected future increases in demand for care. Knowledge of caregiver characteristics and distress determinants among home care recipients with neurological conditions might help alleviate their situation through development of appropriate formal care interventions.

The purpose of this project is to provide a profile of caregivers of home care clients with neurological conditions. Specific objectives are to: (1) describe caregivers of home care clients with neurological conditions living in Canada; (2) describe the amount and type of informal caregiving support they are providing to clients; (3) examine the prevalence of caregiver distress; and (4) identify the association neurological conditions and other client and caregiver characteristics have with caregiver distress.

## Methods

### Study population

The population examined in the present study was comprised of Canadian long stay home care clients (i.e., those expected to be on service for 60 days or longer) in the Winnipeg Regional Health Authority (WRHA) in Manitoba, and in the province of Ontario. These home care clients were targeted in the two jurisdictions since both use the RAI-Home Care (RAI-HC)© [32] as part of routine clinical assessment of long stay clients in their public home care programs. Home care clients with RAI-HC assessment data from January 1, 2003 to December 31, 2010 were eligible for the study. If the client had multiple assessments in that time period, the most recent assessment was used. Only home care clients who had a primary informal caregiver were selected for inclusion. The initial study populations to draw from comprised 36,833 public home care clients in the WRHA and 520,367 public home care clients in Ontario. Once the criterion of having a primary informal caregiver present was applied, the final study sample sizes were 35,964 and 505,551 home care clients, respectively.

The neurological conditions of interest for this study included: Alzheimer’s disease and related dementias (ADRD); multiple sclerosis (MS); amyotrophic lateral sclerosis (ALS); Parkinson’s disease (PD); Huntington disease (HD); epilepsy; muscular dystrophy (MD); cerebral palsy (CP); neurotrauma (both traumatic brain injury (TBI) and spinal cord injury (SCI)); and stroke. Clients who had one or more of the 11 neurological conditions were defined as the neurological study population (*n =* 211,331) and the rest of the sample served as the non-neurological comparison population (*n =* 330,184).

### Data sources

The data sources for the study were the WRHA and the Ontario Association of Community Care Access Centres (OACCAC). The WRHA maintains its own database of RAI-HC assessments for home care clients and the OACCAC manages the RAI-HC database of home care clients within each of the Ontario Local Health Integration Networks (LHINs). All data were subjected to logical coding checks and data cleaning procedures in order to ensure high quality data. These data were anonymized prior to being made available for this research.

RAI-HC assessments are administered by trained regulated health care or social work professionals in both study jurisdictions. The RAI-HC is a standardized assessment that provides data on client characteristics, health status and needs; it is comprised of over 300 assessment items with well established reliability [33]. The RAI-HC identifies five of the 11 neurological conditions of interest in a disease diagnoses pick list section of the assessment (ADRD, MS, PD, Stroke, TBI). The remaining six neurological conditions (ALS, CP, Epilepsy, MD, HD, and SCI) were identified from free text coding of disease diagnoses in the RAI-HC. Neurologists involved in the ideas PNC study checked, agreed, and approved on the methods utilized to identify the neurological conditions.

Two informal caregiver characteristics contained in the RAI-HC were utilized to develop an indicator of caregiver distress. A client’s caregiver was considered to be in distress if one or both of the following was indicated on the assessment: 1) a caregiver is unable to continue in caring activities; 2) the primary caregiver expresses feelings of distress, anger or depression because of caring for the client. These two items are coded by the home care program’s assessors based on clinical judgment informed by their observations and information provided by the client and caregiver. This caregiver distress indicator provides a broader measure of distress in the informal caregiving support system that captures both the feeling of distress and the caregiving situation in distress. This definition of caregiver distress is consistent with previous studies [5, 34].

Data from the RAI-HC also can generate an algorithm, called the Method for Assigning Priority Levels (MAPLe) [34]. The MAPLe algorithm provides a score of 1 to 5 with the client’s level of priority for care increasing with each level. This algorithm can be used to inform choices related to allocation of home care resources and prioritization of clients needing community or facility-based services. It has also been shown to be predictive of caregiver distress in general home care client populations, with rate of caregiver distress increasing with each of the five levels of the MAPLe algorithm [34]. This study provided the opportunity to examine the MAPLe’s association with caregiver distress for clients with neurological conditions specifically.

Additional scales derived from RAI-HC data were utilized in the study to describe the client population and examined for potential associations with caregiver distress. Contributors to caregiver distress identified in the literature aided in selection of applicable RAI-HC scales: the Cognitive Performance Scale (CPS) [35] to examine cognitive impairment; the Activities of Daily Living (ADL) Self-Performance Hierarchy scale [36], and the Instrumental Activities of Daily Living (IADL) Capacity Scale [37] to examine physical demands; the Depression Rating Scale (DRS) [38] as an indicator of depression; and the Changes in Health, End-stage disease and Signs and Symptoms (CHESS), as measure of health instability [39]. Finally, a dichotomous aggressive behaviour indicator was derived based on the presence or absence of any physical abuse or verbal abuse or disruptive/socially inappropriate behaviour, or resisting care. These behaviours are identified by the home care program’s assessors based on clinical judgment informed by their observations and information provided by the clients’ caregivers.

### Statistical methods

This cross-sectional study involved secondary analysis of anonymized data. Descriptive statistics were analyzed to describe and compare the home care client characteristics and caregiver characteristics for each neurological condition of interest, as well as the aggregate neurological and non-neurological study populations. To identify preliminary client and caregiver factors associated with caregiver distress, risk factors were identified through bivariate analysis of RAI-HC items and scales (Chi-square tests), as well as risk factors identified in the literature. Collinearity diagnostics were examined for potential presence of collinearity between independent variables. Logistic regression analysis was employed to identify the risk factors most strongly associated with caregiver distress. All data were analyzed using Statistical Analysis System (SAS) software, version 9.2.

### Ethical aspects

The study protocol was approved by the University of Manitoba Health Research Ethics Board, the University of Waterloo Office of Research Ethics, and the WRHA Research Access and Approval Committee. Permission to access the WRHA RAI-HC data was provided by the WRHA Home Care program. A data sharing agreement between interRAI and OACCAC allowed the research team access to the OACCAC RAI-HC data.

## Results

### Home care clients with neurological conditions

A substantial proportion of home care clients were found to have one or more of the 11 neurological conditions examined in this study (38.8 % in Ontario; 41.9 % in WRHA). Table 1 outlines the number of clients in each of the neurological categories. In both study sites, ADRD is the most prevalent and HD and SCI are the least prevalent conditions.

**Table 1:** Sample Size by Diagnosis & Province

	Ontario (n (%))	Winnipeg RHA (n (%))
ADRD	104,164 (20.6)	8,369 (23.3)
Stroke	85,639 (16.9)	6,230 (17.3)
PD	19,309 (3.8)	1,238 (3.4)
MS	5,947 (1.2)	480 (1.3)
TBI	5,891 (1.2)	438 (1.2)
Epilepsy	5,305 (1.0)	501 (1.4)
CP	1,851 (0.4)	175 (0.5)
ALS	1,433 (0.3)	71 (0.2)
MD	661 (0.1)	82 (0.2)
HD	461 (0.1)	18 (0.1)
SCI	239 (0.05)	27 (0.1)
Any of the 11 neurological conditions	196,269 (38.8)	15,062 (41.9)
None of the 11 neurological conditions	309,282 (61.2)	20,902 (58.1)
Total sample size	505,551	35,964

Table 2 depicts select socio-demographic, clinical and co-morbidity, and home care use characteristics of the clients, by neurological condition, with combined WRHA and Ontario data, and compares this group against clients without any of the 11 conditions (the clients in the “None” column). Clients with CP, MD, MS, and HD were younger clients, with average ages under age 60. The oldest client populations were the ADRD, PD and stroke clients, averaging over 79 years of age. Overall, the largest proportion of clients with neurological conditions were female, but over half of the clients were male for PD, TBI, ALS, MD, and SCI groups. Generally less than half of the clients were married, particularly for clients with CP (9.7 % married), with the exception being clients with ALS, where 68.2 % were married.

**Table 2:** Client Characteristics (Total Study Sample)

Number of clients		ADRD	PD	MS	TBI	Epilepsy	CP	ALS	MD	SCI	HD	Stroke	None	Any
		112,533	20,547	6,427	6,329	5,806	2,026	1,504	743	266	479	91,869	330,184	211,331
Age Group (%)	<65	2.8	5.5	67.7	41.7	47.1	87.5	45.8	72.4	59.4	63.9	9.4	22.5	11.0
	65–74	9.8	18.3	18.8	15.0	16.5	8.0	28.9	14.0	19.2	20.9	15.6	16.3	13.3
	75–84	43.6	49.8	10.9	24.7	23.1	3.7	21.6	10.2	15.4	13.4	40.2	32.0	39.7
	85+	43.9	26.4	2.7	18.7	13.3	0.8	3.7	3.4	6.0	1.9	34.9	30.3	36.1
Mean Age (std)	Years	83.0 (8.5)	79.6 (8.8)	59.0 (13.6)	67.5 (18.2)	64.6 (18.7)	42.8 (17.3)	65.7 (12.1)	51.4 (19.5)	58.6 (18.3)	59.4 (14.5)	79.9 (11.0)	75.6 (15.2)	79.4 (12.5)
Female (%)		63.7	47.3	70.7	49.4	57.5	52.4	45.6	40.0	30.5	57.4	57.7	65.1	60.8
Married (%)		41.0	54.7	53.4	39.0	33.0	9.7	68.2	36.6	42.1^ns^	52.2	42.9	39.2	41.9
CPS Score 2+ (%)		92.7	57.4	28.0	61.9	55.0	50.1	17.0	12.5	6.8	74.7	52.1	19.6	67.4
ADL Hierarchy Score 2+ (%)		44.2	51.8	50.1	33.6	38.5	66.4	71.0	59.9	52.6	52.1	38.2	19.5	40.8
DRS Score 3+ (%)		19.6	18.4	16.2	21.7	16.5	9.9	26.9	10.2	13.5 ^ns^	19.4	16.0	13.9	17.6
CHESS Score 2+ (%)		45.3	40.9	25.6	35.2	29.9	10.4	55.8	24.0	17.7	30.1	38.8	36.8	40.3
Aggressive Behavior (%)		26.3	9.7	3.9	14.7	11.5	12.0	3.1	3.5	4.2	22.3	9.7	3.3	16.5
Diagnoses (%)	CHF	10.3	9.5	3.1	9.3	6.8	1.6	1.8	6.6	4.5	0.6	16.8	13.7	11.8
	CAD	21.8	22.1	8.4	19.8	16.0	2.7	8.4	9.5	10.0	5.1	31.9	22.9	24.2
	Emph/COPD	11.7	11.4	9.0	16.2 ^ns^	17.3 ^ns^	10.6	8.0	11.1	10.3	9.7	16.9 ^ns^	18.8	13.8
	Diabetes	19.8	20.0	12.9	20.2	17.4	6.1	10.0	15.3	15.5	6.9	30.3	24.8	23.1
	Cancer	8.6	9.8	6.0	10.3	9.9	2.8	3.7	3.9	5.8	3.6	11.9	23.7	9.9
Time on Home Care (%)	<1 year	60.8	52.3	40.1	58.9	52.5	37.1	70.0	41.8	47.6	49.2	54.1	66.3	57.1
	1–< 2 years	16.7	17.5	11.4	12.7	13.4	10.7	15.6	12.9	12.9	16.3	14.8	11.7	15.5
	2+ yrs	22.5	30.2	48.5	28.4	34.1	52.2	14.4	45.3	39.5	34.5	31.1	22.0	27.4
Mean Time on Home Care (std)	Years	1.3 (1.9)	1.7 (2.1)	3.5 (4.2)	1.8 (2.9)	2.2 (3.2)	3.9 (4.3)	1.1 (1.9)	3.1 (3.8)	2.7 (3.6)	1.9 (2.4)	1.8 (2.5)	1.3 (2.3)	1.6 (2.4)
Home Care Service	Home Health Aid	60.9	69.7	67.0	54.2 ^ns^	62.7	72.2	58.2	65.2	64.1	59.0	65.6	48.7	62.6
	Home-making	41.4	43.0	40.7	34.4 ^ns^	37.1	39.6	27.6	32.5 ^ns^	34.5 ^ns^	33.1 ^ns^	40.4	32.0	40.5
	Visiting Nurse	24.0	23.4	30.8	26.0	26.5	18.8	37.9	20.9	43.1	14.0	27.2	38.7	25.4

ns - All tests across characteristics for those with the given condition versus those without are statistically significant (*p <* .05) unless otherwise noted

The vast majority of clients with neurological conditions exhibited mild or greater cognitive impairment (67.4 %); this compares to only 19.6 % in the non-neurological client group. The largest proportion of cognitively impaired clients was among ADRD clients (92.7 %) versus the smallest proportion among clients with SCI (6.8 %). Over 40 % of clients in the neurological group exhibited the need for hands-on or greater assistance with ADL activities, double the proportion of clients in the non-neurological group. The largest proportion of clients requiring assistance performing ADLs was found in the ALS group. Signs of possible depression were less prevalent among all home care clients; however, over one quarter of clients with ALS exhibited depressive symptoms. Generally the clients exhibited minimal health instability, but just over 40 % of the neurological group showed some signs of health instability. There was a much higher prevalence of aggressive behaviour among those with neurological conditions (16.5 % vs. 3.3 % among those without); likely due to the larger proportion of clients with ADRD exhibiting aggressive behavior.

Table 2 also demonstrates the complexity of clients with neurological conditions who tend to have multiple comorbid conditions in addition to their neurological condition (e.g., coronary artery disease and diabetes). Overall, the study population had been receiving home care service for over a year on average. The clients in each of the neurological groups were receiving formal home support services (home health aid assistance, homemaking services) to a greater degree than nursing services.

### Caregiver profiles

The characteristics of informal caregivers are presented in Table 3. Similar proportions of primary informal caregivers of clients with and without neurological conditions lived with the client. Just over half of caregivers of persons with neurological conditions lived with the clients, although this was highest for the ALS client population at 80 %. Most primary caregivers were a child/child-in-law of the client, followed by a spouse, but this varied by neurological condition. For example, clients with CP or MD had the highest proportion of other relatives (e.g., parent, sibling) as primary informal caregivers. A secondary informal caregiver was also present for the majority of clients, at a slightly higher rate among clients with neurological conditions.

**Table 3:** Caregiver Characteristics (Total Study Sample)

Number of clients		ADRD	PD	MS	TBI	Epilepsy	CP	ALS	MD	SCI	HD	Stroke	None	Any
		112,533	20,547	6,427	6,329	5,806	2,026	1,504	743	266	479	91,869	330,184	211,331
Primary Informal CG (%)	Lives with client	56.1	64.9	66.6	58.1	56.9 ^ns^	62.6	80.0	71.2	59.4 ^ns^	68.5	58.1	52.2	57.1
Relationship of Primary CG (%)	Child/child-in-law	54.2	41.5	20.1	32.7	27.9	4.1	23.1	13.3	19.6	23.3	50.3	47.2	49.3
	Spouse	33.8	46.8	51.2	35.1	29.2	9.2	63.0	34.9	42.1	49.5	35.6	31.6	35.0
	Other Relative	8.0	7.0	19.4	23.2	31.1	74.9	8.4	44.3	24.4	19.9	8.6	13.0	10.4
	Friend/Neighbor	4.0	4.6	9.2	9.0	11.8	11.8	5.4	7.5	13.9	7.3	5.6	8.2	5.4
Secondary CG (%)	Present	75.8	74.0	63.8	68.1	67.9	67.4	69.5 ^ns^	59.0 ^ns^	54.1	68.5 ^ns^	73.9	68.3	73.9
Primary Informal CG: Areas of Help (%)	ADLs	53.0	59.5	57.6	48.2	49.0	61.4	75.5	62.2	51.9	59.8	51.5	39.3	51.8
	IADLs	92.3	92.3	89.4 ^ns^	87.7	87.5	86.1	95.3	91.1 ^ns^	88.4 ^ns^	90.6 ^ns^	91.6	88.4	91.5
Informal Help – Last 7 days (%)	0–7 h	16.3	15.0	20.9	23.7	27.5	29.1	7.0	20.7	31.4 ^ns^	19.5	18.2	26.4	18.2
	8–14 h	20.0	18.3	19.6	21.5	20.8	12.7	11.5	16.3	19.3 ^ns^	15.6	22.4	29.2	21.3
	15–21 h	24.1	24.8	25.4	22.6	21.2	17.0	23.4	23.3	22.1 ^ns^	20.3	26.0	26.2	25.1
	22+ Hours	39.6	41.8	34.1	32.3	30.5	41.2	58.1	39.7	27.2 ^ns^	44.6	33.4	18.3	35.5
Mean (std) Extent of Informal Help - Last 7 Days	Hours	28.2 (29.6)	28.3 (27.5)	24.0 (24.5)	24.9 (28.9)	24.2 (28.9)	31.2 (33.7)	40.2 (33.4)	27.8 (28.0)	20.9 ^ns^ (24.6)	29.3 (29.1)	24.2 (25.9)	16.3 (19.0)	25.6 (27.5)
Client or CG feel client better living elsewhere (%)	Client Only	1.0	2.0	3.6	3.1	2.5 ^ns^	3.5	2.3	2.6	4.5	1.3	2.0	2.4	1.7
	Caregiver Only	32.4	12.9	4.5	11.7	10.4 ^ns^	6.0	4.9	2.6	1.9	18.0	12.0	4.9	20.4
	Client and Caregiver	15.2	19.4	10.5	13.4	12.3 ^ns^	8.3	12.8	8.6	9.0	27.8	14.4	10.4	14.7
Caregiver Distress (%)		35.3	29.5	19.2 ^ns^	25.6	20.7	19.7 ^ns^	29.7	19.0 ^ns^	16.5 ^ns^	36.7	22.8	13.4	28.0

ns - All tests across characteristics for those with the given condition versus those without are statistically significant (*p <* .05) unless otherwise noted

Nearly all primary informal caregivers were providing clients with assistance in Instrumental Activities of Daily Living (IADLs), and again, this was slightly more prevalent among those with neurological conditions. Assistance with ADLs was less prevalent in general, and within each condition (51.8 % in the neurological group overall). More hours of informal care in a week were provided to clients with neurological conditions. This was particularly notable among clients with ALS, averaging over 40 h per week.

Caregiver distress was twice as prevalent among caregivers of clients with neurological conditions (28.0 % vs. 13.4 % for those caring for people without). Higher than average prevalence rates for caregiver distress were found among clients with HD, ADRD, ALS, and PD. Conversely, caregivers of clients with SCI showed the lowest proportion of distress at 16.5 %. A relatively small proportion of primary caregivers of clients without neurological conditions felt that the client would be better off living elsewhere (15.3 %); this was true for 35.1 % of caregivers of clients with the neurological conditions. A much higher proportion of caregivers of clients with neurological conditions felt the client would be better off in another living environment.

### Risk factors for caregiver distress

The risk factors associated with the risk of caregiver distress are summarized in Table 4. Given the focus on 11 neurological conditions, the unadjusted odds ratios associated with caregiver distress for each condition are presented in the table for comparison of changes when entered into a full model with factors significantly associated with caregiver distress. In unadjusted logistic regression analysis, 6 of the 11 conditions proved to be related to caregiver distress. Caregivers of ADRD clients had the greatest odds of being distressed followed by HD clients. In unadjusted models, MS, epilepsy, CP, MD, and SCI conditions were not significantly associated with caregiver distress. However, when entered into a fully adjusted logistic regression model, epilepsy and SCI remained non-significant but PD and ALS also become unrelated to caregiver distress. HD had the highest association with caregiver distress among the neurological conditions (OR = 1.60), and CP and MD become significantly associated with caregiver distress, both associated with over 30 % increased odds. The remaining neurological conditions remained significant in the adjusted model, but ADRD, MS, and TBI had only modest associations with distress (OR ranged from 1.10 to 1.16). In the adjusted model, stroke became protective for caregiver distress (OR = 0.95).

**Table 4:** Final Logistic Regression Model for Characteristics Associated with Caregiver Distress

Client/Caregiver Characteristic	Unadjusted				Adjusted			
	OR	95 % CI		P value	OR	95 % CI		P value
		Lower	Upper			Lower	Upper	
Male (ref = female)					1.31	1.29	1.33	<.0001
Age 65–74 (Ref = < 65)					1.27	1.23	1.30	<.0001
Age 75–84					1.47	1.43	1.51	<.0001
Age 85+					1.44	1.40	1.48	<.0001
Primary caregiver co-resides					1.70	1.67	1.73	<.0001
Informal hours 1 to 7 (Ref = 0)					1.06	1.00	1.12	0.0485
Informal hours 8 to 14					1.63	1.54	1.73	<.0001
Informal hours 15 to 21					2.05	1.94	2.18	<.0001
Informal hours 22+					2.65	2.50	2.80	<.0001
MAPLe mild (Ref = MAPLe low)					1.24	1.18	1.30	<.0001
MAPLe moderate					1.67	1.61	1.73	<.0001
MAPLe high					2.07	2.00	2.15	<.0001
MAPLe very high					2.50	2.39	2.61	<.0001
ADL hierarchy 1+					1.22	1.20	1.24	<.0001
IADL capacity 1 (Ref = 0)					1.14	1.06	1.23	0.0009
IADL capacity 2					1.29	1.20	1.38	<.0001
IADL capacity 3+					1.42	1.33	1.52	<.0001
Alzheimer’s or related dementia	3.21	3.16	3.26	<.0001	1.16	1.14	1.19	<.0001
Multiple Sclerosis	1.00	0.94	1.06	1.00	1.15	1.08	1.24	<.0001
Traumatic Brain Injury	1.43	1.35	1.51	<.0001	1.10	1.03	1.18	0.0032
Huntington Disease	2.44	2.03	2.94	<.0001	1.60	1.30	1.98	<.0001
Stroke	1.34	1.32	1.36	<.0001	0.95	0.93	0.97	<.0001
Muscular Dystrophy	0.98	0.81	1.17	0.8075	1.32	1.08	1.61	0.0070
Cerebral Palsy	0.98	0.88	1.09	0.6675	1.35	1.19	1.52	<.0001
Parkinson’s Disease	1.85	1.80	1.91	<.0001	1.03	0.99	1.07	NS
Epilepsy	1.06	1.00	1.13	0.0734	1.00	0.93	1.07	NS
ALS	1.83	1.64	2.04	<.0001	1.11	0.98	1.25	NS
Spinal Cord Injury	0.79	0.57	1.08	0.1415	1.34	0.94	1.91	NS
DRS 1 to 2 (Ref = 0)					1.51	1.48	1.54	<.0001
DRS 3+					2.16	2.11	2.20	<.0001
CPS 1 to 2 (Ref = 0)					1.44	1.41	1.47	<.0001
CPS 3 to 6					1.49	1.44	1.53	<.0001
CHESS 1 to 2 (Ref = 0)					1.14	1.11	1.16	<.0001
CHESS 3 to 5					1.26	1.23	1.29	<.0001
Client openly expresses conflict/anger					1.49	1.46	1.53	<.0001
Economic tradeoffs					1.68	1.60	1.76	<.0001
Worsening behaviour					1.94	1.89	1.99	<.0001
Conditions make health pattern unstable					1.48	1.45	1.50	<.0001
Hallucinations or delusions					1.11	1.07	1.15	<.0001
Overall Increase in Care Needs					1.43	1.40	1.45	<.0001
Daily home support hours last 7 days					0.99	0.98	0.99	<.0001
Daily nursing hours last 7 days					0.85	0.83	0.87	<.0001
Ontario (compared to Manitoba)					0.74	0.72	0.77	<.0001

The largest associations with caregiver distress were with the amount of informal care hours provided in a week and the MAPLe algorithm. The odds of caregiver distress increased with each categorical increase in informal hours; caregivers of clients receiving 22 h or more of informal care in the last week were 2.65 times more likely to experience distress. Similar increments were found with the MAPLe algorithm, where caregivers of clients at the very high priority level for MAPLe had 2.50 times the odds of having distressed caregivers after adjusting for all other factors in the model.

Higher risk of distress was identified when the client showed signs of potential clinical depression (DRS 3+; OR = 2.16); worsening behaviour (OR = 1.94); co-resided with the caregiver (OR = 1.70); when clients openly expressed conflict or anger with family/friends (OR = 1.49); and when clients had made economic trade-offs in the previous month for purchasing required medical (e.g., medications) or environmental support (e.g., home heating) (OR = 1.68).

Greater odds of caregiver distress were also found when clients had impairments in cognition (OR up to 1.49 for moderate or greater impairment, CPS 3-6) and IADL capacity (OR up to 1.42); were not independent in performance of ADLs performance (OR = 1.22); had a condition that made their health pattern unstable (OR = 1.48) or current higher health instability (CHESS 3-5; OR = 1.26); experienced hallucinations or delusions (OR = 1.11); or were experiencing a decline/deterioration in status (OR = 1.43). Male clients and older clients had higher odds of distressed caregivers, associated with 30 % (males) and up to 44 % (clients age 85+) increased odds respectively.

Provision of formal home care services provided a protective effect. For every one hour increase in formal home support hours in a week, the odds of caregiver distress decreased (OR = 0.99). Formal nursing support indicated an even larger impact on decreasing the odds of caregiver distress with every hour increase (OR = 0.85). Provincial jurisdiction also showed an impact, with caregivers in Ontario having lower odds of distress (OR = 0.74).

### MAPLe algorithm and caregiver distress for neurological populations

**Fig. 1:**
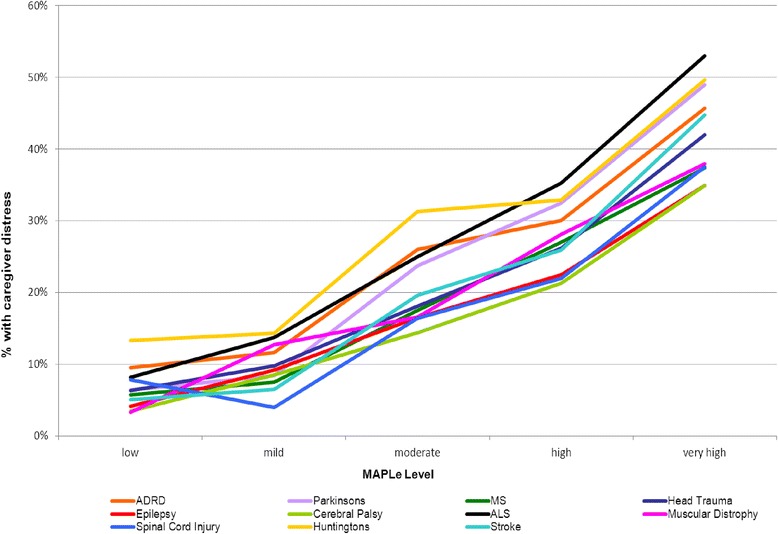
Caregiver Distress by MAPLe Score and Diagnosis (Total Study Sample). Figure 1 illustrates that the percentage of distressed caregivers becomes greater with each increase in the five MAPLe priority levels, for each specific neurological condition

**Fig. 2:**
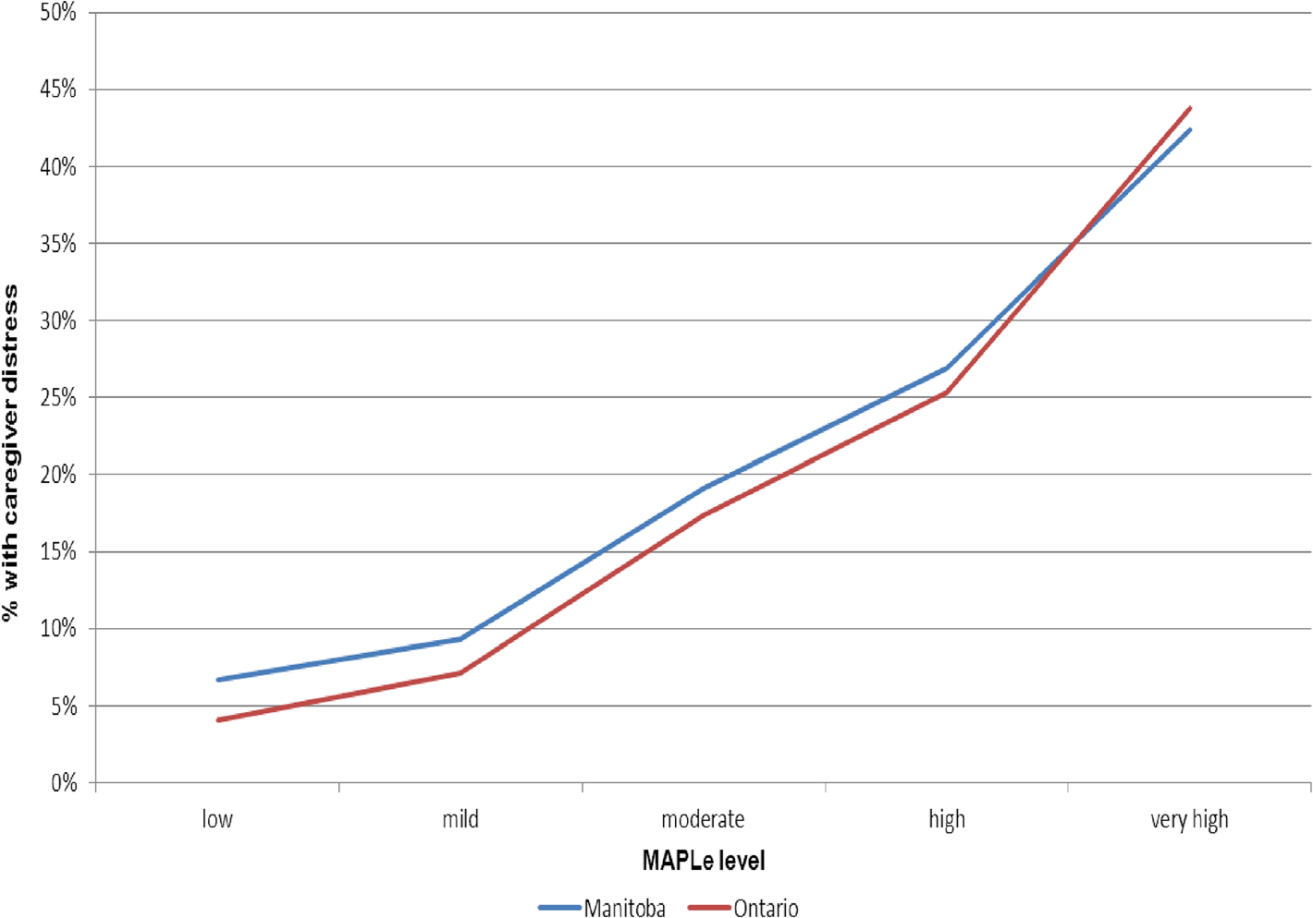
Caregiver Distress by MAPLe Score and Region. Figure 2 illustrates a nearly identical pattern of increase in caregiver distress with each increasing MAPLe level when the WRHA and Ontario data are examined separately

The MAPLe decision support algorithm was designed to be used to inform home care professionals decisions about prioritization of access to community and facility services based on various combinations of functional and clinical need indicators. The logistic regression analysis identified that the MAPLe algorithm had one of the strongest associations with caregiver distress. Figure 1 illustrates that the percentage of distressed caregivers became greater with each increase in the five MAPLe priority levels, for each specific neurological condition. However, while the pattern of increase was the same, the percentage of caregivers exhibiting distress was varied within groups. For example, at the highest MAPLe priority level, only 35 % of caregivers of clients with CP or epilepsy exhibited distress while 53 % of caregivers of clients with ALS exhibited distress. Figure 2 illustrates a nearly identical pattern of increase in caregiver distress with each increasing MAPLe level when the WRHA and Ontario data were examined separately.

## Discussion

Neurological conditions were highly prevalent among home care clients and family members provide a great deal of support to persons with neurological conditions living in the community. Indeed, the majority of care received comes from informal sources, and the provision of that care can have a negative impact on informal caregivers. The large study population provided by census level home care data allowed for the identification and comparison of clients and caregivers within a greater span of neurological conditions than has been previously seen in the literature. This unique approach provides a more robust understanding of the caregiving experience for clients with neurological conditions in general, but more specifically through comparison of the specific conditions.

Client characteristics varied by neurological condition, which resulted in varied caregiver characteristics and care provision profiles. Yet some similar patterns in caregivers and caregiving emerged among the 11 neurological conditions. The majority of caregivers lived with the client and nearly all provided assistance with IADLs, but to a lesser extent with ADLs. For all conditions, the caregivers provided more hours of informal help and a higher proportion experienced caregiver distress than among caregivers of clients without neurological conditions. These similarities in caregiving patterns aid in identifying considerations for caregiver support strategies. However, the heterogeneity within home care clients with neurological conditions produced variation in the relationship of the caregiver to the client and prevalence of caregiver distress. This differential caregiver experience suggests some caregiver populations may be less vulnerable to distress and there is the need to customized support strategies for different neurological groups. The complexity of clients with neurological conditions indicates multicomponent support strategies are required for caregivers.

The present study replicated what was previously found in the neurological literature [e.g., 8, 10, 13, 14, 20-22, 27]. Caregiver distress increased as amount of time providing care increased; with signs of client depression, behavior, and cognitive impairment; and with client limitations in ADLs and IADLs. Clients’ clinical characteristics had a stronger association with caregiver distress than the simple presence or absence of a neurological condition. Nonetheless, after adjustment for these characteristics, a diagnosis of Huntington disease produced a strong association with caregiver distress, as did cerebral palsy and muscular dystrophy, to a lesser extent. The finding suggests there are facets related to certain neurological conditions and distress that the risk factors in this study did not address.

An important contribution of this research was identification of the significant relationship between the MAPLe algorithm and caregiver distress among home care clients in general, and for the specific neurological conditions. Higher MAPLe scores produced some of the highest odds ratios for caregiver distress in the study, and prevalence of distress increased as the MAPLe score increased for all of the neurological conditions studied. This finding is not surprising given that the MAPLe algorithm is calculated based on multiple domains and interactions, such as ADL impairment, cognitive impairment, wandering, behavior problems, falls, risk for institutionalization, and environmental problems [34], and provides a global indication of complexity and priority for care. This finding validates that the MAPLe algorithm can be utilized in home care programs to identify care needs and priority for service among home care clients with neurological conditions as well as at risk caregiver populations for this important and growing segment of home care clients.

Few neurological studies have examined the role of formal home care provision and its relationship with caregiver distress. Kumamoto and colleagues (2006) [40] found that home care service reduced burden among family caregivers of clients with dementia. The present study was able to augment evidence in this area while examining the different impact of formal home support and nursing support. This study did find a pronounced protective effect between formal home care service provision and caregiver distress. As hours of formal care increased, odds of caregiver distress decreased. This effect was strongest for formal nursing care. Examining additional home care services for their impact on caregiver distress, such as social workers and occupational and physical therapists, would be beneficial in future research. Such information could aid in identifying a service team best able to reduce caregiver distress.

The finding that caregivers of home care clients in Ontario were at reduced risk of distress compared to the WRHA points to potential differences in home care provision in the two jurisdictions. There may be services in Ontario provided to clients and caregivers not measured in this study that resulted in lessening caregiver distress. Further exploration of this finding might be helpful in informing support strategies nationally. Conversely, there may be potential jurisdictional differences in detecting caregiver distress. Further investigation into this result is merited, since under detection of caregiver distress could lead to lack of support to caregivers in need.

Informal caregivers support the health care system by assuming a substantial proportion of care and cost to persons in need. This research has direct relevance to caregiver support initiatives and strategies by providing evidence on informal caregivers who support persons with complex neurological and health needs, whose need for home care services often begins at a much younger age (depending on the condition). Knowledge of the caregiver characteristics and risk factors for distress assists in improving the caregiving experience through development of appropriate support strategies and interventions, which directly benefits the caregiver, the care recipient, and health care systems [30].

### Limitations

The study has several limitations. It was not feasible to examine all possible contributing factors to caregiver distress in this exploratory research. Additional details on caregivers, such as length of time caregiving, age, and gender, were not available in the data. Nor was it possible to identify types of support strategies/services caregivers may be accessing. This additional information would enhance the profile of caregivers of clients with neurological conditions and potential modifiers of distress. Also, this study focused solely on identifying individual risk factors that contributed most significantly to caregiver distress. Interactions between individual client characteristics were not examined due to the large number of variables involved in this study. However the research did focus on the MAPLe algorithm, which in itself is a complex combination of and interactions between client characteristics. Results identified the MAPLe algorithm is among the most significant risk factors for distress.

Lastly, future research in this area may benefit from alternative operationalizations of caregiver distress. The caregiver distress outcome utilized in this study was a dichotomous variable that only detected presence or absence of distress. It would be beneficial to use a measure of distress that can identify level so that the neurological conditions or client characteristics that are related to the greatest levels of caregiver distress can be more accurately identified. In addition, it may be informative to explore utilization of an indicator that captures only explicitly reported feelings of distress from the caregiver due to the caregiving role as opposed to the two criteria utilized in this research.

## Conclusions

Neurological conditions are common among home care clients and a significant proportion of informal caregivers providing care to these clients experience distress. Caregiver distress was more prevalent among clients with neurological conditions than clients without such conditions. Amount of time providing informal care and the overall complexity of the client based on the MAPLe algorithm showed the greatest association with caregiver distress. These factors were more important to caregiver distress than the diagnosis of a neurological condition. Nonetheless, certain neurological conditions, such as Huntington disease, cerebral palsy, or muscular dystrophy, were associated with caregiver distress, even after adjusting for other significant risk factors. The complexity of clients with neurological conditions suggests the need for multicomponent support strategies for informal caregivers.

## Abbreviations

ADLs: Activities of daily living
ADRD: Alzheimer’s disease and related dementias
ALS: Amyotrophic lateral sclerosis
CHESS: Changes in Health, End-stage disease and Signs and Symptoms
CP: Cerebral palsy
CPS: Cognitive Performance Scale
DRS: Depression Rating Scale
HD: Huntington disease
IADLs: Instrumental activities of daily living
ideas PNC: Innovations in Data, Evidence and Applications for Persons with Neurological Conditions
LHINs: Local Health Integration Networks
MAPLe: Method for Assigning Priority Levels
MD: Muscular dystrophy
MS: Multiple sclerosis
NHCC: Neurological Health Charities of Canada
OACCAC: Ontario Association of Community Care Access Centres
PD: Parkinson’s disease
PHAC: Public Health Agency of Canada
RAI-HC©: RAI-Home Care
SAS: Statistical Analysis System
SCI: Spinal cord injury
TBI: Traumatic brain injury
WHO: World Health Organization
WRHA: Winnipeg Regional Health Authority
